# Monthly anomaly database of atmospheric and oceanic parameters in the tropical Atlantic ocean

**DOI:** 10.1016/j.dib.2022.107969

**Published:** 2022-02-17

**Authors:** H.L. Varona, F. Hernandez, A. Bertrand, M. Araujo

**Affiliations:** a Laboratory of Physical, Coastal and Estuarine Oceanography (LOFEC). Department of Oceanography (DOCEAN). Center for Technology and Geosciences (GTG). Federal University of Pernambuco. Recife-PE. Brazil; b Center for Studies and Tests in Risk and Environmental Modeling (CEERMA). Federal University of Pernambuco. Recife-PE. Brazil; c Institut de Recherche pour le Développement (IRD), LEGOS, Univ Toulouse, CNRS, CNES, Toulouse, France; d MARBEC, Univ Montpellier, CNRS, Ifremer, IRD, Sète, France; e Brazilian Research network on Global Climate Change (Rede CLIMA), São José dos Campos, SP, Brazil

**Keywords:** MARDAO dataset, TAAD dataset, Anomaly, Tropical Atlantic, Climate change

## Abstract

The Tropical Atlantic Ocean Database and Monthly Anomalies of River Discharge on Atlantic Ocean datasets encompass the monthly anomalies of a variety of physical, biogeochemical parameters from the tropical Atlantic Ocean and the monthly anomalies of river runoff in the Atlantic Ocean and its adjacent seas. The parameters used as the base for the computation of anomalies come from the TROPFLUX, GPCP, ASCAT, SODA, GODAS, DASK, SeaWiFS, OAFLUX, WAVEWATCH III, NOAA/ESRL 20th Century Reanalysis, GLOBAL_REANALYSIS_BIO_001_029, GLOBAL_REANALYSIS_BIO_001_033, OCEANCOLOUR_GLO_OPTICS_L4_REP_OBSERVATIONS_009_081, OSCAR, SMOS, MODIS-Aqua, CO2_Flux, and GRDC datasets. Several of the anomaly data are redundant, but come from different data sources making comparative studies possible. For ease of use, both datasets are provided in NetCDF format, CF convention. These datasets include 18 files in NetCDF format, which facilitates its handling due to the diversity of freeware tools that exist and are structured in two-, three- and four-dimensional grids. All these anomalies can be useful to oceanographers, meteorologists, ecologists and other researchers for studies of climate variation in the tropical Atlantic Ocean. These datasets are hosted at https://www.seanoe.org/data/00718/82962/ and https://data.mendeley.com/datasets/pn5b35vn6s/1.

## Specifications Table

**Table:** 

Subject	Oceanography, Atmospheric Sciences
Specific subject area	Physical, chemical and biological oceanography. Atmospheric surface fluxes.
Type of data	River stations (Time series, 1807–2021 monthly period). Tri-dimensional and fourth-dimensional grids (1947–2019 monthly period).
How the data were acquired	The data were obtained through the computation of anomalies from the existing datasets: TROPFLUX (Air-Sea Fluxes for the Global Tropical Oceans) GPCP (Global Precipitation Climatology Project) ASCAT (Advanced Scatterometer) WAVEWATCH3 model SODA (Simple Ocean Data Assimilation) GODAS (Global Ocean Data Assimilation System) DASK (Data Assimilation System of KIOST (Korea Institute of Ocean Science and Technology)) SeaWiFS (Sea-viewing Wide Field-of-view Sensor) OAFLUX (Objectively Analyzed air-sea Heat Fluxes) NOAA/ESRL 20th Century Reanalysis (NOAA-CIRES-DOE 20th Century Reanalysis) GLOBAL_REANALYSIS_BIO_001_029 (Copernicus Marine Service) GLOBAL_REANALYSIS_BIO_001_033 (Copernicus Marine Service) OCEANCOLOUR_GLO_OPTICS_L4_REP_OBSERVATIONS_009_081 (Copernicus Marine Service) OSCAR (Ocean Surface Current Analysis in Real-time) SMOS (Soil Moisture and Ocean Salinity) MODIS-Aqua (Moderate Resolution Imaging Spectroradiometer) CO2_Flux (Optimized air-sea CO2 flux for the Global Ocean) GRDC (Global Runoff Data Centre from Bundesanstalt für Gewässerkunde)
Data format	NetCDF embedding metadata
Parameters for data collection	All the parameters were obtained through time series with frequency of monthly means and distributed geospatially in two-dimensional and three-dimensional grids.
Description of data collection	The original datasets were downloaded directly from the official websites
Data source location	Runoff river stations at Atlantic Ocean is limited by 113°W – 44°E/51°S – 70°N and grids at Tropical Atlantic Ocean is limited by 65°W – 20°E/30°S – 30°N.
Data accessibility	The collection of NetCDF files is published at the following address: Title: Tropical Atlantic Anomaly Database (TAAD). Repository name: SEANOE Data identification number: 10.17882/82962 Direct URL to data: https://www.seanoe.org/data/00718/82962/ And Title: MARDAO: Monthly Anomalies of River Discharge on Atlantic Ocean. Repository name: Mendeley Data Data identification number: 10.17632/pn5b35vn6s.1 Direct URL to data: https://data.mendeley.com/datasets/pn5b35vn6s/1 Tools for the creation of NetCDF files and for the calculation of anomalies: Title: mNC: A tool for Oceanographers and Meteorologists to easily create their NetCDF files using Matlab.
	Repository name: Zenodo Data identification number: 10.5281/zenodo.5572749 Direct URL to data: https://zenodo.org/record/5572749 And Title: CalcPlotAnomaly: Matlab function set for the calculation and plotting of anomalies. Repository name: Zenodo Data identification number: 10.5281/zenodo.5576889 Direct URL to data: https://zenodo.org/record/5576889

## Value of the Data

• The main objective of this work was to gather a series of products offering a reliable representation of past reality in the tropical Atlantic. Either by choosing gridded products based directly in-situ and satellite observations, or by choosing products based on numerical simulations and modelling approaches, constrained to realism by data assimilation (the so-called reanalysis) or other technics. The data presented here encompass the monthly anomalies of physical, chemical and biological parameters in the tropical Atlantic Ocean. This dataset can be useful for any researcher that may need these data for further analyses or interpreting physical, biogeochemical or biological patterns or processes of oceanographic and atmospheric parameters in the tropical Atlantic Ocean. It is relevant to study changes in ocean climate through statistical studies. It can also be used as a reference when compared to fully simulated representations of ocean and atmospheric dynamics during the past decades, like the IPPC and CMIP6 coupled simulations. It can also be used for visualization for official uses, decision-makers, general public, education and outreach activities.
• This dataset is made up of multiple NetCDF files using the CF convention, sharing similar time coordinates, making it easy to share. It is extremely easy to use and does not require any prior processing.

## 1 Data Description

**Fig. 1:**
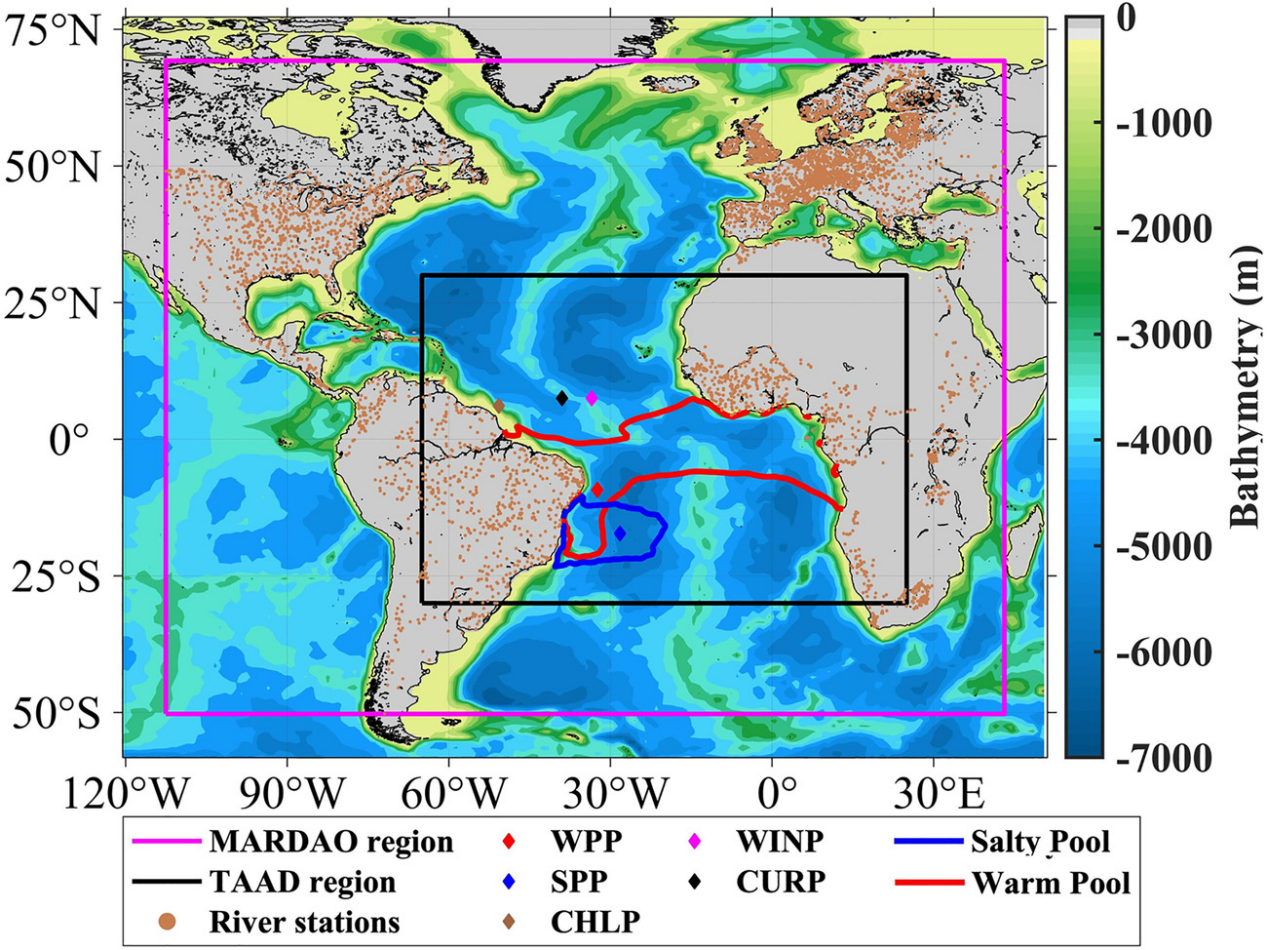
Geographical location of the MARDAO and TAAD dataset regions. Geographical location of WPP (Warm Pool Point), SPP (Salty Pool Point), CHLP (Chlorophyll Concentration Point), CURP (Surface Currents Point) and WINP (Surface Winds Point), and river stations.

These datasets present runoff anomalies at stations on all rivers discharging freshwater into the Atlantic Ocean and adjacent seas (MARDAO dataset) and anomalies of surface fluxes and physical, chemical and biological parameters at different ocean depths in the Tropical Atlantic Ocean (TAAD dataset) Fig. 1 shows the geographical boundaries of each dataset, the position of all river runoff stations. In the TAAD dataset there are redundant parameter anomalies (e.g., water temperature, salinity, ocean currents, winds, chlorophyll concentration, etc.), this is to facilitate researchers to make comparative studies of monthly climatic variations, the points WPP, SPP, CHLP, CURP and WINP will be used to show such comparisons (Fig. 1, Table 1).

**Table 1:** Geographical locations of the MARDAO and TAAD regions and the coordinates of the WPP, SPP, CHLP, CURP and WINP points.

Description	Name of the regionor point	Geographical location
MARDAO region	-	112.5°W – 43.5°E/50.5°S – 69.5°N
TAAD region	-	65°W - 20°E/30°S – 30°N
Warm Pool region	Northeastern Brazil	44.25°W - 31.25°W/11.25°S - 1.25°S
Salty Pool region	Northeast and Southeast Brazil	40.25°W - 26.25°W/22.25°S - 12.25°S
Warm Pool Point	WPP	32.4°W, 9.25°S
Salty Pool Point	SPP	28.25°W, 17.25°S
Chlorophyll Concentration Point	CHLP	50.75°W, 6.1°N
Current Speed Point	CURP	39°W, 7.5°N
Wind Speed point	WINP	33.5°W, 7.5°N

All anomaly data files are in NetCDF format, CF convention, the Monthly Anomalies of River Discharge on Atlantic Ocean dataset (MARDAO) contains only one anomaly data file (located in the https://seanoe.org/ repository), while the Tropical Atlantic Anomaly Database (TAAD) contains 19 zip files (Located in the repository https://data.mendeley.com/), which contain 20 files in NetCDF format, the **87798.zip** file contains 2 NetCDF files because the anomalies of the marine current components are separated from the rest Table 2 shows the details of the original datasets used to calculate the monthly anomalies, such as the center that produces it, the periods, the spatial resolution of each grid and the filename that each file has in the repository.

**Table 2:** Original dataset from which the monthly anomalies were calculated. ESSO/INCOIS - Indian National Centre for Ocean Information Services. ECMWF - European Centre for Medium-Range Weather Forecasts. PSL **-** Physical Sciences Laboratory. DASK - Data Assimilation System of KIOST (Korea Institute of Ocean Science & Technology). OCO - NOAA Office of Climate Observations. CCDD - Climate Change Data and Detection. CMEMS - Copernicus Marine Environment Monitoring Service. NIES - National Institute for Environmental Studies. ESR - Earth & Space Research. ESRL - Earth System Research Laboratories. BfG - Bundesanstalt für Gewässerkunde. * Means that it contains two netcdf files.

Original dataset source	Producer center	Reference	Time period	Frequency	Spatial resolution	Filename
TROPFLUX	ESSO/INCOIS	[1]	Jan/1979 - Dec/2017	Daily	1°	87822.zip
GPCP	NOAA /University of Maryland	[2]	Oct/1996 - Dec/2020	Daily	1°	87800.zip
ASCAT	NOAA	[3]	Mar/2007 - Nov/2018	Daily	0.25°	87796.zip
WAVEWATCH III	NOAA	[4]	Jan/1997 - May/2019	3 hours	1.25° x 1°	87823.zip
SODA	ECMWF	[5]	Jan/1980 - Dec/2017	Monthly	0.5	87828.zip
GODAS	PSL/NOAA	[6]	Jan/1980 - Sep/2020	Monthly	1° x 0.333°	87809.zip
DASK	KIOST	[7]	Jan/1947 - Dec/2012	Monthly	1° x 1° - 1/3°	87798.zip*
SeaWiFS	NASA	[8]	Sep/1997 - Dec/2010	Weekly	0.25°	87820.zip
OAFLUX	NOAA (OCO – CCDD)	[9]	Jan/1958 - Jun/2019	Daily	1°	87801.zip
NOAA/ESRL 20th Century Reanalysis	NOAA	[10]	Jan/1836 - Nov/2015	Daily	1°	87802.zip
GLOBAL REANALYSIS BIO 001_029	CMEMS	[11]	Jan/1993 - Nov/2019	Monthly	0.25°	87795.zip
GLOBAL REANALYSIS BIO 001_033	CMEMS	[12]	Jan/1998 - May/2019	Weekly	0.25°	87799.zip
OCEANCOLOUR GLO OPTICS L4 REP OBSERVATIONS 009_081	CMEMS	[13]	Sep/1997 - Feb/2020	Monthly	0.041667°	87805.zip
OSCAR	ESR	[14]	Oct/1992 - Nav/2020	5 days	0.3333°	87819.zip
SMOS	ESA	[15]	Jan/2010 - Nov/2020	4 days	0.259366°	87821.zip
MODIS-Aqua	NASA	[16]	Aug/2002 - Jun/2020	Daily	0.041667°	87803.zip
CO2 Flux	NIES	[17]	Jan/1980 - Dec/2009	Monthly	1°	87797.zip
GRDC (Global Runoff Data Centre)	BfG	[18]	Jan/1806 – Mar/2021	Monthly	-	anomGRDC-Monthly.nc

Table 3 shows the description of all physical, chemical and biological parameters for which anomalies were calculated. In this Table are listed the name of each parameter, to which the suffix **_anom** was added, with the exception of the parameter **runoff_mean** of the MARDAO dataset (this parameter contains the original runoff data at all stations of each river). In addition to the name of each parameter, the unit, the type of grid and the original set to which they belong are included. In the TAAD dataset the data are organized in two types of grids, the 3D type grids, which are the parameters that are found at the ocean surface or at a fixed depth, therefore, they depend on longitude, latitude and time. The 4D type grids are organized similarly to the 3D type grids, but in addition to longitude, latitude and time they also depend on depth. In the case of the MARDAO dataset the anomaly data are organized in time series for each station.

**Table 3:** Parameter Description. * Means parameter added to dataset. WW3 - WAVEWATCH III and GFS models. GFS - Global Forecast System. 20CRv3 - NOAA/ESRL 20^th^ Century Reanalysis. 001_029 - means product GLOBAL_REANALYSIS_BIO_001_029. 001_033 – means product GLOBAL_REANALYSIS_BIO_001_033. 009-081 – means product OCEANCOLOUR_GLO_OPTICS_L4_REP_OBSERVATIONS_009_081.

Parameter name	Description	Unit	Grid type	Original dataset source
lhf_anom	Latent heat flux (downward is the positive direction)	W m^−2^	3D	TROPFLUX
lwr_anom	Net surface longwave radiation (downward is the positive direction)	W m^−2^	3D	TROPFLUX
netflux_anom	Net surface heat flux (downward is the positive direction)	W m^−2^	3D	TROPFLUX
q2m_anom	Specific humidity at 2m	g kg^−1^	3D	TROPFLUX
shf_anom	Sensible heat flux (downward is the positive direction)	W m^−2^	3D	TROPFLUX
sst_anom	Sea surface temperature	°C	3D	TROPFLUX
swr_anom	Short wave radiation	W m^−2^	3D	TROPFLUX
t2m_anom	Air temperature at 2 m	°C	3D	TROPFLUX
tau_anom	Wind stress magnitude	N m^−2^	3D	TROPFLUX
taux_anom	Zonal wind stress	N m^−2^	3D	TROPFLUX
tauy_anom	Meridional wind stress	N m^−2^	3D	TROPFLUX
ws_anom	Wind speed at 10m	m s^−1^	3D	TROPFLUX

precip_anom	Daily precipitation rate at ocean surface	mm day^−1^	3D	GPCP

uwnd_anom	Zonal wind speed	m s^−1^	3D	ASCAT
vwnd_anom	Meridional wind speed	m s^−1^	3D	ASCAT
wspd_anom*	Wind speed	m s^−1^	3D	ASCAT

ugrdsfc_anom	Surface zonal wind speed	m s^−1^	3D	WW3
vgrdsfc_anom	Surface meridional wind speed	m s^−1^	3D	WW3
perpwsfc_anom	Surface primary wave mean period	s	3D	WW3
htsgwsfc_anom	Surface sig height of wind waves and swell	m	3D	WW3
wspdfc_anom*	Wind speed	m s^−1^	3D	WW3

temp_anom	Seawater potential temperature	°C	4D	SODA
salt_anom	Seawater salinity	psu	4D	SODA
ssh_anom	Sea surface height above geoid	m	3D	SODA
mlt_anom	Mixed layer depth determined by temperature criteria	m	3D	SODA
mlp_anom	Depth of potential density mixed layer	m	3D	SODA
mls_anom	Mixed layer depth determined by salinity criteria	m	3D	SODA
net_heating_anom	Surface ocean heat flux coming through coupler and mass transfer	W m^−2^	3D	SODA
prho_anom	Potential density referenced to 0 dbar	Kg m^−3^	4D	SODA
u_anom	Seawater zonal velocity	m s^−1^	4D	SODA
v_anom	Seawater meridional velocity	m s^−1^	4D	SODA
taux_anom	Surface downward zonal stress	N m^−2^	3D	SODA
wt_anom	Vertical current velocity	m s^−1^	4D	SODA
tauy_anom	Surface downward meridional stress	N m^−2^	3D	SODA

thflx_anom	Total downward heat flux at ocean surface (downward is positive)	W m^−2^	3D	GODAS
sltfl_anom	Salt flux at ocean surface	g cm^−2^ s	3D	GODAS
sshg_anom	Sea Surface Height Relative to Geoid	m	3D	GODAS
dbss_obil_anom	Isothermal layer depth	m	3D	GODAS
dbss_obml_anom	Mixed layer depth	m	3D	GODAS
uflx_anom	Momentum flux, zonal component	N m^−2^	3D	GODAS
vflx_anom	Momentum flux, meridional component	N m^−2^	3D	GODAS
salt_anom	Salinity	psu	4D	GODAS
ucur_anom	Zonal component of the ocean current	m s^−1^	4D	GODAS
vcur_anom	Meridional component of the ocean current	m s^−1^	4D	GODAS
spd_anom*	Current speed	m s^−1^	4D	GODAS
dzdt_anom	Vertical velocity of the sea current	m s^−1^	4D	GODAS
pottmp_anom	Potential temperature	K	4D	GODAS

co2_anom	CO_2_ flux, positive downward	Mole m^−2^ s	3D	CO2_Flux

sfc_hflux_pme_anom	Heat flux (relative to 0°C) from Precipitation minus Evaporation transfer of water across ocean surface	watts m^−2^	3D	DASK
river_anom	Mass flux of river (runoff + calving) entering ocean	Kg m^−3^ * m s^−1^	3D	DASK
mld_anom	Mixed layer depth determined by density criteria	m	3D	DASK
temp_anom	Potential temperature	°C	4D	DASK
salt_anom	Salinity	psu	4D	DASK
u_anom	Zonal component of the ocean current	m s^−1^	4D	DASK
v_anom	Meridional component of the ocean current	m s^−1^	4D	DASK
spd_anom*	Current speed	m s^−1^	4D	DASK

sss_anom	Sea surface salinity	psu	3D	SMOS

chlor_a_anom	Chlorophyll concentration, oci algorithm	mg m^−3^	3D	MODIS-Aqua
sst_anom	Sea surface temperature	°C	3D	MODIS-Aqua

chla_anom	Chlorophyll concentration	mg m^−3^	3D	SeaWiFS

evapr_anom	Evaporation rate at ocean surface	mm day^−1^	3D	OAFLUX

ucurr_anom	Zonal component of the ocean current	m s^−1^	3D	OSCAR
vcurr_anom	Meridional component of the ocean current	m s^−1^	3D	OSCAR

spd_anom*	Current velocity	m s^−1^	3D	OSCAR
tminc_anom	Minimum temperature at 2m	°C	3D	20CRv3
tmaxc_anom	Miximum temperature at 2m	°C	3D	20CRv3
airc_anom	Air temperature al 2m	°C	3D	20CRv3
dlwrf_anom	Downward longwave radiation flux at ocean surface (downward is the positive direction)	W m^−2^	3D	20CRv3
dswrf_anom	Downward solar radiation flux at ocean surface (downward is the positive direction)	W m^−2^	3D	20CRv3
lhtfl_anom	Latent heat net flux at ocean surface (downward is the positive direction)	W m^−2^	3D	20CRv3
pevpr_anom	Potential evaporation rate at ocean surface	W m^−2^	3D	20CRv3
prate_anom	Precipitation rate at ocean surface	Kg m^−2^ s	3D	20CRv3
rhum_anom	Relative humidity at 2m	Kg Kg^−1^	3D	20CRv3
shtfl_anom	Sensible heat net flux at ocean surface (downward is the positive direction)	W m^−2^	3D	20CRv3
shum_anom	Specific humidity at 2m	Kg Kg^−1^	3D	20CRv3
ulwrf_anom	Upward longwave radiation flux at ocean surface	W m^−2^	3D	20CRv3
uswrf_anom	Upward solar radiation flux at ocean surface	W m^−2^	3D	20CRv3
uwnd_anom	Wind zonal velocity at 10m	m s^−1^	3D	20CRv3
vwnd_anom	Wind meridional velocity at 10m	m s^−1^	3D	20CRv3
wspd_anom*	Wind velocity	m s^−1^	3D	20CRv3
hflb_anom*	hflb = lhtfl - shtfl (downward is the positive direction)	W m^−2^	3D	20CRv3
spco2_anom	Surface partial pressure of carbon dioxide in sea water	Pa	3D	001_029
o2_anom	Mole concentration of dissolved molecular oxygen in sea water	mmol m^−3^	4D	001_029
chla_anom	Mass concentration of chlorophyll a in sea water	mg m^−3^	4D	001_029
no3_anom	Mole concentration of nitrate in sea water	mmol m^−3^	4D	001_029
po4_anom	Mole concentration of phosphate in sea water	mmol m^−3^	4D	001_029
phyc_anom	Mole concentration of phytoplankton expressed as carbon in sea water	mmol m^−3^	4D	001_029
si_anom	Mole concentration of silicate in sea water	mmol m^−3^	4D	001_029
ph_anom	Sea water PH reported on total scale	-	4D	001_029
nppv_anom	Net primary production of biomass expressed as carbon per unit volume in sea water	mg m^−3^ day^−1^	4D	001_029

fe_anom	Mole concentration of dissolved iron in sea water	mmol m^−3^	4D	001_029
depth_epi_anom	Sea water epipelagic layer depth	m	3D	001_033
depth_lmeso_anom	Sea water lower mesopelagic layer depth	m	3D	001_033
depth_umeso_anom	Sea water upper mesopelagic layer depth	m	3D	001_033
mnkc_epi_anom	Mass concentration of epipelagic micronekton expressed as wet weight in sea water	g m^−2^	3D	001_033
mnkc_lhmmeso_anom	Mass concentration of lower highly migrant mesopelagic micronekton expressed as wet weight in sea water	g m^−2^	3D	001_033
mnkc_lmeso_anom	Mass concentration of lower mesopelagic micronekton expressed as wet weight in sea water	g m^−2^	3D	001_033
mnkc_lmmeso_anom	Mass concentration of lower migrant mesopelagic micronekton expressed as wet weight in sea water	g m^−2^	3D	001_033
mnkc_umeso_anom	Mass concentration of upper mesopelagic micronekton expressed as wet weight in sea water	g m^−2^	3D	001_033
mnkc_ummeso_anom	Mass concentration of upper migrant mesopelagic micronekton expressed as wet weight in sea water	g m^−2^	3D	001_033
zooc_anom	Mass concentration of zooplankton expressed as carbon in seawater	g m^−2^	3D	001_033

CDM_anom	Colored dissolved and detrital organic materials - Mean of the binned pixels	m^−1^	3D	009_081
KD490_anom	Diffuse attenuation coefficient - Mean of the binned pixels	m^−1^	3D	009_081
RRS443_anom	Fully normalized remote sensing reflectance at 443 nm - Mean of the binned pixels	sr^−1^	3D	009_081
SPM_anom	Inorganic suspended particulate matter in sea water - Mean of the binned pixels	g m^−3^	3D	009_081
ZSD_anom	Secchi disk depth - Mean of the binned pixels	m	3D	009_081

runoff_mean	Monthly river runoff	m^3^ s^−1^	Station	GRDC
runoff_anom	Monthly anomaly river runoff	m^3^ s^−1^	Station	GRDC

Note that the product GLOBAL_REANALYSIS_BIO_001_033 was removed from the CMEMS catalog, and replaced in 2021 by the product GLOBAL_MULTIYEAR_BGC_001_033. Both are based on the SEAPODYM ecosystem model, the former at the ¼° resolution with one week frequency estimates. It is forced by weekly means of Mercator Ocean circulation model (without assimilation), ERA-Interim atmospheric fields, and primary production issued from the CMEMS derived GLOBCOLOUR surface chlorophyll concentration.

Only evaporation has been taken from the OAFLUX dataset because the rest of the parameters coincide with those of the TROPFLUX dataset. For all parameters the missing data is represented by NaN (Not a Number), in the metadata of each parameter **_FillValue** and **missing_value** are assigned to NaN. The time reference for the MARDAO dataset is ``days since 1700-01-01 00:00'' and for the TAAD dataset is ``days since 1900-1-1 00:00:00''.

In the MARDAO dataset in addition to the data file containing the runoff anomalies at all stations of all rivers there are 3 directories, the **figures** directory containing the figures **fig_RiverStationsMap.jpeg** (Map with the representation of all stations) and **fig_AmazonRiverAnomaly.jpeg** (figure showing the runoff anomalies at 3 stations of the Amazon River), the **matlab** directory containing the script **get_and_plot_data.m** script showing how to use this dataset and finally the **Stationlist** directory containing several files (in CSV, DBF, HTML, LibreOffice Calc and Microsoft Excel formats) with the ID of all stations, the name of each station as well as the name of the rivers to which each station belongs and the data owner.

**Fig. 2:**
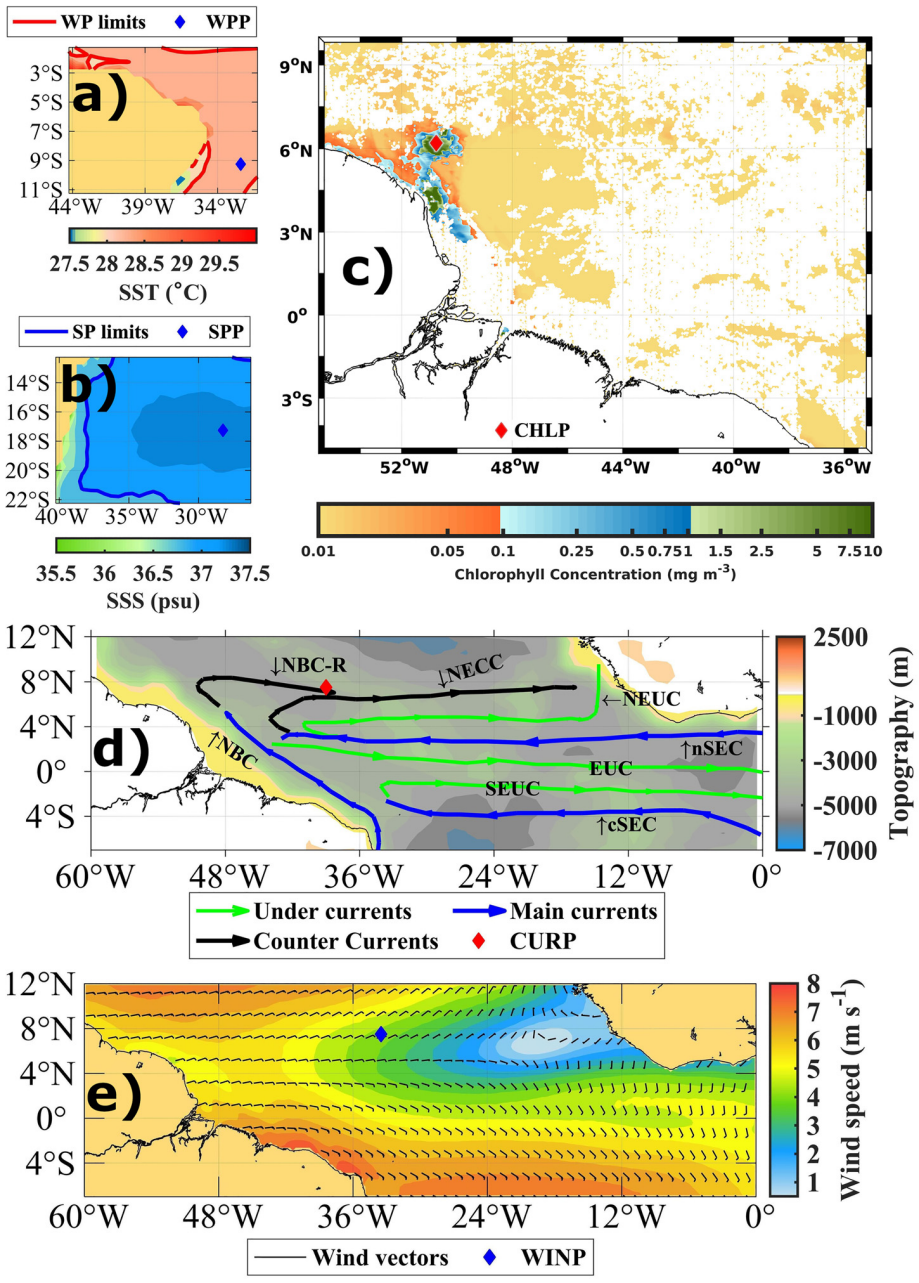
Geographical location of the points chosen for the comparison of anomalies; a) Sea Surface Temperature, March 1980 (SODA dataset); b) Sea Surface Salinity, annual mean (SODA dataset); c) Mean weekly climatology of chlorophyll concentration, first week of October, Moderate-Resolution Imaging Spectroradiometer (MODIS), near the mouth of the Amazon River (adapted from [19]); d) Sea currents in the tropical Atlantic Ocean (adapted from [20]); NBC (North Brazil Current); NBC-R (North Brazil Current North Brazil Current Retroflection); cSEC (central branch of South Equatorial Current); nSEC (north branch of South Equatorial Current); NECC (North Equatorial Countercurrent); EUC (Equatorial Undercurrent); SEUC (South Equatorial Undercurrent); NEUC (North Equatorial Undercurrent). e) Surface winds in the tropical Atlantic Ocean, annual mean (ASCAT dataset).

**Fig. 3:**
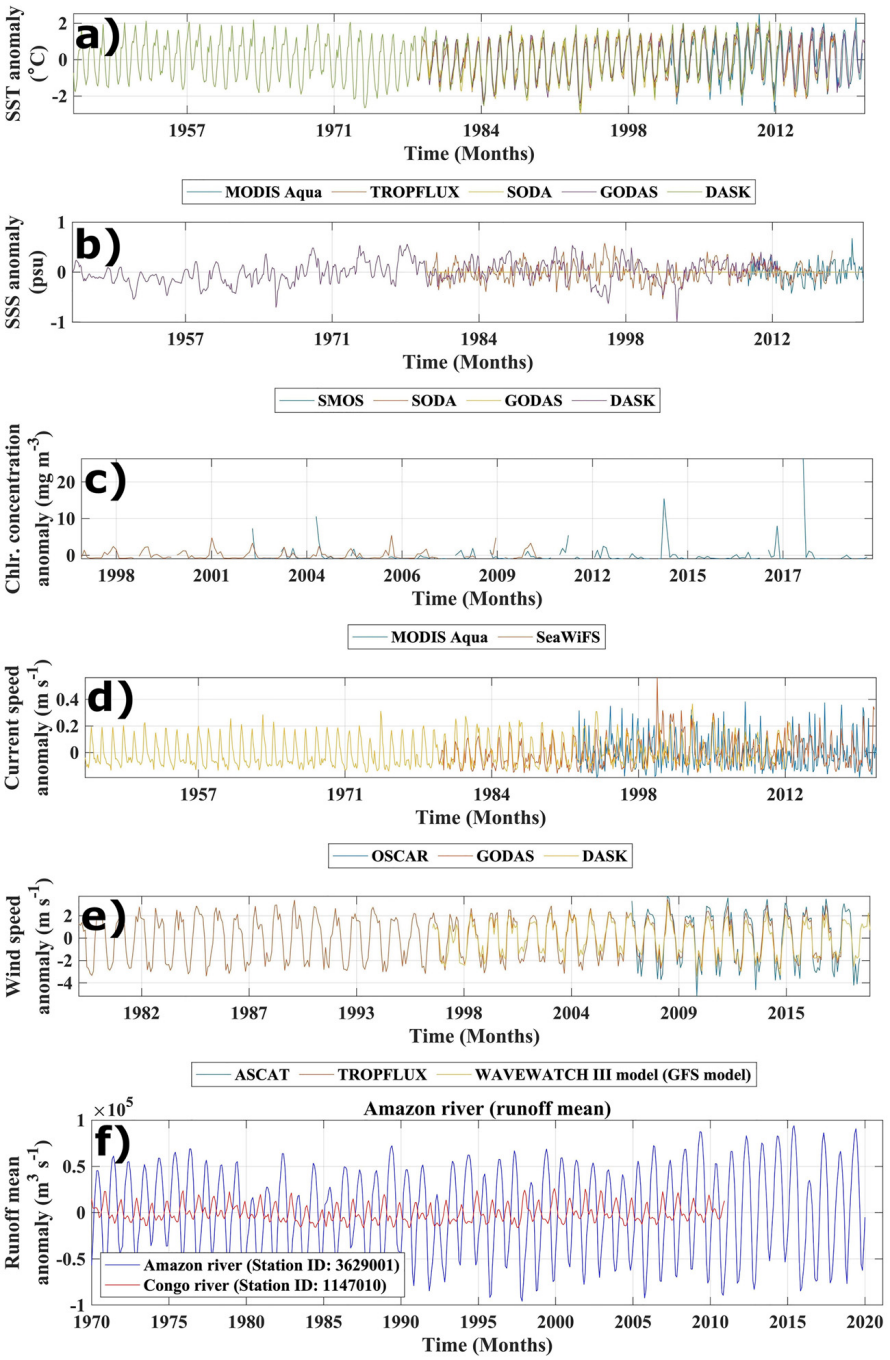
Comparison of similar parameter anomalies with different data sources; a) Sea Surface Temperature anomalies at WPP point; b) Sea Surface Salinity anomalies at SPP point; c) Chlorophyll concentration anomalies at CHLP point; d) Surface current velocity anomalies at CURP point; e) Surface wind velocity anomalies at WINP point; f) Comparison of runoff anomalies of the Amazon (Station ID 3629000) and Congo (Station ID 1147010) rivers, MARDAO dataset.

As mentioned earlier, in the TAAD dataset encompasses redundant parameters to facilitate comparative studies of anomalies according to different data sources. As an example of the value of this we have chosen several points to show a comparison between anomalies according to different datasources (see Fig. 2 and Table 1 for the locations of these points). The WPP was chosen due to the presence of a Warm Pool that appears in that region from February to April or May (Fig. 2a), the location of the SPP is due to the presence of a permanent Salty Pool in that region (Fig. 2b), at the CHLP the chlorophyll concentration varies according to the Amazon River plume (Fig. 2c, adapted from [19]), at the CURP is where the retroflexion of the North Brazil Current (NBC) feeds the north equatorial countercurrent (Fig. 2d, adapted from [20]) and finally the WINP is chosen because this is the place where the Intertropical Convergence Zone (ITCZ) shows maximum variability (Fig. 2e) Fig. 3. shows comparisons of anomalies between similar parameters (the term “similar parameter” means that they are the same parameters but obtained from different datasets, see Table 4) with different data sources (Sea Surface Temperature, Sea Surface Salinity, Chlorophyll concentration, current velocity and surface wind), also showing a comparison between runoff anomalies (MARDAO dataset) at station 3629000 (Amazon River) and station 1147010 (Congo River).

**Table 4:** Similar parameters based on your source dataset.

Similar parameters	Datasets
sst_anom*	TROPFLUX, MODIS-Aqua, 20CRv3
sss_anom*	SMOS
salt_anom	SODA, GODAS, DASK
ssh_anom, sshg_anom	SODA, GODAS
temp_anom, pottmp_anom	SODA, GODAS, DASK
ws_anom, wspd_anom, wspdfc_anom	TROPFLUX, ASCAT, WW3, 20CRv3
precip_anom, prate_anom	GPCP, 20CRv3
lwr_anom, dlwrf_anom	TROPFLUX, 20CRv3
swr_anom, dswrf_anom	TROPFLUX, 20CRv3
lhf_anom, lhtfl_anom	TROPFLUX, 20CRv3
shf_anom, shtfl_anom	TROPFLUX, 20CRv3
uwnd_anom, ugrdsfc_anom	ASCAT, WW3, 20CRv3
vwnd_anom, vgrdsfc anom	ASCAT, WW3, 20CRv3
netflux_anom, net_heating_anom	TROPFLUX, SODA
spd_anom	GODAS, DASK, OSCAR
u_anom, ucurr_anom	SODA, GODAS, DASK, OSCAR
v_anom, vcurr_anom	SODA, GODAS, DASK, OSCAR
chlor_a_anom, chla_anom	MODIS-Aqua, 001_029
t2m_anom, airc_anom	TROPFLUX, 20CRv3
wt_anom, dzdt_anom	SODA, GODAS
q2m_anom, shum_anom	TROPFLUX, 20CRv3
mlp_anom, mld_anom	SODA, DASK
taux_anom	TROPFLUX, SODA
tauy_anom	TROPFLUX, SODA

⁎ It is also included in the datasets that have potential temperature and salinity.

## 2 Experimental Design, Materials and Methods

The data from the original datasets that were used to calculate the anomalies had different frequencies: every 3 hours, every 6 hours, daily and monthly. The MARDAO and TAAD datasets are presented with monthly anomalies so first the monthly averages were calculated for the datasets that had a frequency lower than monthly. In the case of precipitation of the GPCP dataset, the data were organized in daily precipitation, so the accumulated precipitation in each month was calculated.

Once all the grids (TAAD dataset) and stations (MARDAO dataset) had monthly frequency, the anomalies were calculated using the Matlab script set called **CalcPlotAnomaly**, the creation of all the NetCDF files was done using the Matlab script set called **mNC**. Once these processes were completed, all metadata were added using the **nco** software.

## Ethics Statements

Not applicable.

## CRediT Author Statement

**H. L. Varona:** Conceptualization, Methodology, Validation, Formal analysis, Investigation, Data curation, Writing- Original draft, Visualization. **F. Hernandez:** Methodology, Validation, Visualization, Writing- Reviewing and Editing. **A. Bertrand:** Conceptualization, Methodology, Validation, Visualization, Writing- Reviewing and Editing. **M. Araujo:** Conceptualization, Visualization, Writing- Reviewing and Editing, Supervision, Project administration, Funding acquisition.

## Declaration of Competing Interest

The authors declare that there is no conflict of interest regarding the publication of this article. The authors also declare that they have no known competing financial interests or personal relationships which have, or could be perceived to have, influenced the work reported in this article.

## Data Availability

MARDAO: Monthly Anomalies of River Discharge on Atlantic Ocean (Original data) (Mendeley Data) Tropical Atlantic Anomaly Database (TAAD) (Original data) (Seanoe)
